# The miR-183/*Taok1* Target Pair Is Implicated in Cochlear Responses to Acoustic Trauma

**DOI:** 10.1371/journal.pone.0058471

**Published:** 2013-03-05

**Authors:** Minal Patel, Qunfeng Cai, Dalian Ding, Richard Salvi, Zihua Hu, Bo Hua Hu

**Affiliations:** 1 Center for Hearing and Deafness, State University of New York at Buffalo, Buffalo, New York, United States of America; 2 Center for Computational Research, New York State Center of Excellence in Bioinformatics & Life Sciences, Department of Ophthalmology, Department of Biostatistics, Department of Medicine, State University of New York at Buffalo, Buffalo, New York, United States of America; University of Salamanca- Institute for Neuroscience of Castille and Leon and Medical School, Spain

## Abstract

Acoustic trauma, one of the leading causes of sensorineural hearing loss, induces sensory hair cell damage in the cochlea. Identifying the molecular mechanisms involved in regulating sensory hair cell death is critical towards developing effective treatments for preventing hair cell damage. Recently, microRNAs (miRNAs) have been shown to participate in the regulatory mechanisms of inner ear development and homeostasis. However, their involvement in cochlear sensory cell degeneration following acoustic trauma is unknown. Here, we profiled the expression pattern of miRNAs in the cochlear sensory epithelium, defined miRNA responses to acoustic overstimulation, and explored potential mRNA targets of miRNAs that may be responsible for the stress responses of the cochlea. Expression analysis of miRNAs in the cochlear sensory epithelium revealed constitutive expression of 176 miRNAs, many of which have not been previously reported in cochlear tissue. Exposure to intense noise caused significant threshold shift and apoptotic activity in the cochleae. Gene expression analysis of noise-traumatized cochleae revealed time-dependent transcriptional changes in the expression of miRNAs. Target prediction analysis revealed potential target genes of the significantly downregulated miRNAs, many of which had cell death- and apoptosis-related functions. Verification of the predicted targets revealed a significant upregulation of *Taok1,* a target of miRNA-183. Moreover, inhibition of miR-183 with morpholino antisense oligos in cochlear organotypic cultures revealed a negative correlation between the expression levels of miR-183 and *Taok1,* suggesting the presence of a miR-183/*Taok1* target pair. Together, miRNA profiling as well as the target analysis and validation suggest the involvement of miRNAs in the regulation of the degenerative process of the cochlea following acoustic overstimulation. The miR-183/*Taok1* target pair is likely to play a role in this regulatory process.

## Introduction

The loss of sensory cells in the cochlea due to acoustic overstimulation is irreversible because these cells are completely differentiated and do not regenerate once they die. The resultant hair cell loss can be further exacerbated by exposure to ototoxic drugs or by aging [1]–[5]. To prevent hair cell loss from occurring, understanding the molecular mechanisms involved in regulating the sensory cell death associated with acoustic trauma is critical for the development of effective treatments.

Acoustic overstimulation induces sensory cell degeneration via complex pathways with apoptotic and necrotic phenotypes [6]–[12]. Multiple apoptosis-related proteins have been identified during noise-induced sensory cell degeneration [13]–[19]. Transcriptional changes in apoptosis-related genes have also been found following acoustic trauma [20]. These observations illustrate the complexity of cochlear responses to acoustic trauma. However, the molecular mechanisms responsible for the changes in the expression of these genes are not clear.

More recently, microRNAs (miRNAs) have been found to play an essential role in regulating cell degeneration [21]–[23]. miRNAs, small 20–22 nucleotide molecules, represent a new class of non-coding RNA genes. miRNAs regulate cellular functions by modulating mRNA expression levels [24]. Increasing evidence suggests the involvement of miRNAs in the transcriptional regulation of apoptosis-related genes [25]–[33]. Therefore, modulation of miRNA function represents a novel and potentially powerful strategy for regulating gene expression with significant clinical potential for disease prevention.

In the auditory system, investigations of miRNA functions have been mainly focused on their roles in inner ear development [34]–[38]. The role of miRNAs in noise-induced cochlear pathogenesis is yet to be established. Given the finding of strong apoptotic activity in noise-traumatized cochleae, we hypothesized that miRNAs are critically involved in cochlear pathogenesis after acoustic trauma. To test this hypothesis, we performed an experimental study with the following three specific aims: (1) to profile the constitutive expression of miRNAs in normal and noise-damaged rat cochlear sensory epithelia, (2) to use bioinformatic analysis to identify potential mRNA targets of the miRNAs and (3) to experimentally verify the predicted targets of the miRNAs.

Here, we show the constitutive expression of miRNAs in both normal and noise-traumatized cochlear sensory epithelia, many of which have not been previously reported in cochlear tissues. Noise exposure significantly decreased the expression of a subset of miRNAs. Using bioinformatic analysis, we predicted the potential mRNA targets of these miRNAs, many of which had roles in the regulation of cell death and apoptosis. Experimental verification of the predicted genes revealed miR-183/*Taok1*as a possible miRNA-mRNA target pair. This prediction was confirmed by the regulation of miR-183 expression using morpholino oligos. In summary, the current investigation implicates miRNAs in cochlear responses to acoustic trauma.

## Materials and Methods

### Ethics Statement

All procedures involving the use and care of animals were reviewed and approved by the Institutional Animal Care and Use Committee of the State University of New York at Buffalo.

### Animals

Sprague Dawley rats (220–300 gm, 2–3 months, male and female, Charles River Laboratories, Wilmington, MA) with normal hearing sensitivity evaluated with the auditory brainstem response (ABR) were used in this study. The animals were randomly assigned to 2 groups: either noise-exposed groups or control groups. The noise groups contained animals sacrificed at either 2 h post-noise exposure (2 h group, n = 4) or 1 day post-noise exposure (1 d group, n = 8). Each noise-exposed animal was paired with a control animal, which received identical treatment except for the noise exposure. Additional control animals (n = 6) were used for immunostaining and western blot experiments.

Sprague-Dawley rat pups (n = 7, postnatal day 3, male and female, Charles River Laboratories) were used for organotypic culture studies.

All procedures involving the use and care of animals were reviewed and approved by the Institutional Animal Care and Use Committee of the State University of New York at Buffalo.

### Noise exposure

Awake-rats from the 2 h and 1 d groups were exposed to a broadband continuous noise (1–7 kHz) at 120 dB SPL (re 20 µPa) for 2 h. This level of noise was chosen because it is capable of inducing permanent hearing loss and sensory cell apoptosis [20], [39] The noise signal was generated with a real-time signal processor (RP2.1, TDT, Alachua, Fl), routed through an attenuator (PA5, TDT) and a power amplifier (Crown XLS 202, Harman International Company) connected to a loud speaker (NSD2005–8, Eminence). The speaker was suspended directly above the animal holding cage. The noise level at the position of the animal’s head in the sound field was calibrated using a sound level meter (Larson and Davis 800 B, Depew, NY), a preamplifier (Larson and Davis, model 825) and a ½” condenser microphone (Larson and Davis, LDL 2559). Rats were individually exposed to the noise in the holding cage.

### Auditory Brainstem Response Test

Prior to noise exposure, 2 h and 1 d after noise exposure, ABRs were measured individually for the right and left ear to determine the hearing sensitivity of each animal from all groups. Animals were lightly anesthetized by intraperitoneal injection of a mixture of ketamine (87 mg/kg) and xylazine (3 mg/kg). ABR thresholds were measured by placing stainless steel needle electrodes subdermally over the vertex (noninverting electrode) and posterior to the stimulated and non-stimulated ear (inverting electrode and ground electrode) of the animal. The acoustic stimuli were 5, 10, 20, 30 and 40 kHz tone bursts (0.5 msec rise/fall Blackman ramp, 1 msec duration, alternating phase) presented at the rate of 21/second. Stimuli were generated digitally using a D/A converter (RP2.1, TDT, 100 kHz sampling rate) and fed to a programmable attenuator (PA5, TDT), amplifier (SA1, TDT) and closed-field loudspeaker (CF1, TDT). The electrode outputs were delivered to an amplifier (RA4LI and RA4PA; TDT) and then to a medusa base station (RA16BA, TDT). TDT software (BioSig) controlled the auditory evoked response averaging system. Responses were filtered (100–3000 Hz), amplified and averaged for 250 stimulus presentations using TDT hardware and software. These responses were then stored and displayed on a computer. Stimulus levels were decreased in 5 dB steps until the ABR response disappeared. The ABR thresholds were defined as the lowest intensity that reliably elicited a detectable waveform of the response.

### Euthanasia and Harvesting of Cochlear Sensory Epithelia

For miRNA gene array expression analysis, the animals (n = 4 for each noise group and each control group) were decapitated under CO_2_-gas anesthesia upon completion of the final ABR test at either 2 h or 1 d post-noise exposure. One cochlea from each subject was used for miRNA array gene expression analysis. This cochlea was immediately perfused through the round window with an RNA stabilization reagent (RNAlater, Qiagen, Valencia, CA) and then carefully dissected to remove the bony wall and the lateral wall tissue in the RNAlater reagent under a dissection microscope. Two-thirds of the cochlear sensory epithelium containing the top and middle portions of the sensory epithelium was collected for total RNA isolation. The other cochlea from each animal was used for pathological analysis and fixed with 10% buffered formalin.

For mRNA gene expression analysis, the animals (n = 4 for 1 d noise group and n = 4 for control group) were decapitated under CO_2_-gas anesthesia upon completion of the final ABR test at 1 d post-noise exposure. Both cochleae from each subject were used for mRNA gene expression analysis. The cochleae were immediately perfused through the round window with an RNA stabilization reagent (RNAlater, Qiagen, Valencia, CA) and then carefully dissected to remove the bony wall and the lateral wall tissue in RNAlater reagent under a dissection microscope. Two-thirds of the cochlear sensory epithelium containing the top and middle portions of the sensory epithelium was collected for total RNA isolation as described in a below section.

### Pathological Analysis

Morphological examination of the sensory epithelia was performed for both the normal (n = 4 cochleae) and the noise-traumatized cochleae (n = 4 cochleae for each 2 h and 1 d group). Propidium iodide (Invitrogen, Carlsbad, CA), a nuclear stain, was used to label the sensory epithelia. The animals were sacrificed as detailed in the above section. The cochleae were harvested, fixed with 10% buffered formalin and then dissected to collect the organs of Corti. The collected tissues were incubated in the staining solution (5 µg/ml in 10 mM phosphate buffered saline, PBS) for 10 min, washed with PBS, and mounted on slides containing antifade medium (ProLong™ antifade kit, Invitrogen). The criteria for identification of damaged cells have been described in previous publications [9], [11], [19]. Briefly, cells with condensed or fragmented nuclei were considered apoptotic cells. Viable cells were those with the normal nuclear size and shape.

### Isolation of Total RNA

Total RNA was isolated from either the cochlear sensory epithelium or cochlear organotypic cultures using an RNA isolation kit (RNeasy Plus Mini Kit, Qiagen). The cochlear tissue was physically disrupted using a rotor-stator homogenizer in 150 µl Qiazol lysis buffer for 30 seconds. The solution was incubated for 10 min at room temperature and then 50 µl of chloroform was added. The mixture was shaken vigorously for 15 seconds, incubated for 5 min at room temperature, and then centrifuged (12,000 rpm) for 15 min at 4°C. The upper clear aqueous layer was transferred into a centrifuge tube and combined with 175 µl of 75% ethanol, mixed and passed through a spin column (provided in the Qiagen RNeasy Mini Kit) and centrifuged at 8000 rpm for 20 sec. The solution was washed with the manufacturer-provided buffer three times. Finally, total RNA was collected in 30 µl of RNase free water. The quantity and quality of collected total RNA were evaluated first using a Nanodrop 2000 Spectrophotometer (Thermo Scientific, Wilmington, DE) followed by an Agilent Bioanalyzer 2100 (Agilent Technologies, Santa Clara, CA) using procedures described in our previous publication [39].

### miRNA Expression Analysis Using microRNA Array Cards

Cochlear miRNA expression patterns were analyzed using TaqMan Low Density Rodent microRNA arrays (TLDA) (v. 2.0, A-card, Applied Biosystems, Foster City, CA). This array card contained probes for 378 mature miRNAs, 5 endogenous controls (U6, SnoRNA135, SnoRNA202, U87 and Y1) and a negative control probe (ath-miR-159a). The miRNAs included in this array provide comprehensive coverage of Sanger miRBase and include those miRNAs that have been highly characterized in non-cochlear tissues.

The isolated total RNA was reverse transcribed using Megaplex™ RT Rodent Primers Pool and the TaqMan MiRNA Reverse Transcription Kit (Applied Biosystems). The resulting cDNA template mixture was further used to run the pre-amplification reaction using the TaqMan PreAmp Master Mix and Megaplex PreAmp Primers (Applied Biosystems). Individual pre-amplified products were mixed with TaqMan Gene Expression Master Mix and loaded into each of the eight well ports on the TLDA card. The card was centrifuged twice for 1 min at 1200 rpm and then sealed to prevent contamination. The TLDA cards were run on an ABI 7900HT real time PCR system (Applied Biosystems) for 40 cycles as per manufacturer’s protocol. Four biological replications were performed for each group.

### Bioinformatic Target Analysis

For miRNA target analysis, we used an open source software program, TargetScan mouse version 5.1 [40]–[42] and the database; DAVID [43], [44]. TargetScan was used to predict biological regulatory targets of miRNAs. Once a target gene list for a miRNA was identified, they were input into DAVID, which sorted these targets into functionally related clusters. In our analysis, a “high” level of classification stringency was selected to classify targets into functional clusters as described below. The software uses an enrichment score to depict the importance of functional clusters within individual miRNA target gene lists. Functional clusters with a higher enrichment score were more biologically relevant compared to clusters within the same gene list with a lower enrichment score. This stringency allowed us to predict only highly relevant functional targets within individual miRNA target gene lists.

DAVID was also used to identify significantly enriched GO terms (biological process) from the identified functional clusters. Each term was associated with an EASE score (p value) calculated using a modified Fisher exact test. The lower the EASE score (p-value) the higher the enrichment of GO terms (biological process). Identification of functional clusters and GO terms were performed for each individual miRNA.

### mRNA Expression Analysis

The transcriptional expression levels of *Nfat5, Taok1, Xiap, Map3k2* and *Bach2* were examined to verify the predicted targets of miRNAs identified via bioinformatic analysis. The expression levels of these genes were analyzed using pre-developed TaqMan gene expression primer/probe assays (Applied Biosystems). The isolated total RNAs from the control (n = 4) and the 1 d post-noise exposure groups (n = 4) were reverse transcribed using a High Capacity cDNA reverse transcription kit (Applied Biosystems). qRT-PCR was performed on a MyIQ-two color real time PCR detection system (BioRad, Hercules, CA). Pre-developed *Hprt1, Rplp1* and *Actb* gene expression assays (Applied Biosystems) were used as endogenous controls.

### Immunohistochemistry

Immunohistochemistry was used to examine the expression pattern of Taok1 protein in normal sensory epithelia from 3 rats. After ABR testing, the animals were anesthetized and sacrificed. The cochleae were quickly removed from the skull and fixed with 10% buffered formalin overnight. After dissection in 0.1 M PBS, the organs of Corti were collected and permeabilized with 0.2% Triton X-100 in PBS for 30 min. Specimens were blocked with 10% goat serum in PBS, and incubated overnight at 4°C in a solution containing primary antibody (Taok1 (PSK2), sc-83463, Santa Cruz Biotechnology, Inc., Santa Cruz, CA) at a concentration (1∶200) recommended by the manufacturer. After incubation, the tissues were rinsed with PBS (3×), incubated with a secondary antibody (Alexa Fluor 488-labeled donkey anti-goat antibody for Taok1) for 2 h, and then counterstained with propidium iodide (5 µg/ml in PBS) for 10 min. The tissues were mounted on slides containing antifade medium. Images of immunolabeled tissues were taken with confocal microscopy (Zeiss LSM510, Carl Zeiss Microscopy, Thornwood, NY) using a method that has been reported previously [20], [45] The Taok1 positive cells were identified by green fluorescence (excitation at 499 nm and emission at 519 nm). As a negative control, tissue samples from both normal and noise exposed cochleas were incubated with only secondary antibody during the tissue processing to assess non-specific staining.

### Western Blot

Western blot analysis was performed to identify the presence of Taok1 protein in the normal sensory epithelium from 3 rats. Gapdh was used as a cochlea tissue-specific loading control. Cochlear tissues containing the sensory epithelium and the lateral wall from two cochleae of 1 animal were pooled together to generate 1 sample. The samples were homogenized and lysed in 100 µl RIPA lysis buffer (Santa Cruz Biotechnology Inc) on ice. Centrifugation at 12,000 rpm for 20 min at 4°C pelleted the nuclei and cell debris, leaving the supernatant which was used for western blotting. The protein samples were then denatured and separated under reducing conditions by electrophoresis in a NuPAGE Novex 4–12% Bus-Tris Gel (Invitrogen) at 125 V for 2 h. The SeeBlue Plus2 Pre-stained standard and MagicMark XP were used as protein markers (Invitrogen). After electrophoresis, the proteins were transferred onto a 0.2 µm PVDF transfer membrane (Invitrogen) for 2 h at 30V. Once the proteins were transferred onto the membranes, they were blocked using 5% non-fat powdered milk in 1 × Tris-buffered saline (TBS) with 0.05% TWEEN-20 (TBSt) followed by incubation with the primary antibody (Taok1 (PSK2), sc-83463, Santa Cruz Biotechnology, Inc.) or the tissue-specific loading control primary antibody (Gapdh, ABS16, EMD Millipore, Billerica, MA) eat 4°C overnight. Then, the membranes were washed in 1×TBSt (3×), incubated with a secondary antibody (donkey anti-goat-IgG-horseradish peroxidase (HRP) or donkey anti-rabbit-IgG-HRP, Santa Cruz Biotechnology Inc.) and visualized using the Chemiluminescent substrate for HRP (Thermo Fisher Scientific, Waltham, MA).

### Cochlear Organotypic Cultures

Cochlear organotypic cultures were prepared from neonatal SASCO Sprague–Dawley rat cochleae at postnatal day 3 as described previously [46], [47]. Briefly, the cochlea was removed and the organ of Corti transferred onto rat tail type I collagen gel in basal medium Eagle containing 2% sodium carbonate. A 15 µL drop of the collagen solution was placed on the surface of a 35 mm culture dish and allowed to gel for approximately 30 min. Afterwards, 2 ml of culture medium (0.01 g/ml bovine serum albumin, 1% Serum-Free Supplement [Sigma I-1884], 2.4% of 20% glucose, 0.2% penicillin G, 1% BSA, 2 mM glutamine, 95.4% of 1× BME) was added to the dish. The cultures were maintained in an incubator at 37°C and 5% CO_2_ for 24 h. After 7 h, fresh medium alone or fresh medium containing Endo-Porter transfection reagent (6 µM) along with either fluorescein-conjugated standard control morpholino or miR-183* morpholino oligo (5 µM, Gene-Tools, Philomath, OR) was added.

Morpholino antisense oligo sequences were: Fluoresceinated Scrambled Control (5′-CCTCTTACCTCAGTTACAATTTATA-3′) and miR-183* (5′- ACAGTGAATTCTACCAGTGCCATAC-3′ Fluorescein).

After a 24 h incubation period, the tissues were further processed either for confocal microscopy or for qRT-PCR as described above. For confocal microscopy, the cochlear tissue was fixed with 10% phosphate buffered formalin for 4 h and then rinsed with 0.01 M PBS. The tissues were mounted on glass slides in glycerin, coverslipped, and examined using a confocal microscope (Zeiss, LSM510; absorption: 501 nm, emission: 524 nm) to confirm the success of the oligo delivery. For qRT-PCR, the explants were collected and processed for isolation of total RNA as described in the above sections.

### Data Analysis

ABR thresholds obtained pre-, 2 h and 1 d post-noise exposure at 5, 10, 20, 30 and 40 kHz frequencies were compared using a two-way ANOVA with post hoc Tukey’s test.

For the miRNA array data analysis, a cut-off C_T_ value of 34 was selected and any target with the C_T_ value equal to or lower than 34 was considered as detected. The NormFinder software algorithm (Andersen et al., 2004) was used to identify stable endogenous control genes for expression level normalization of the miRNAs (ΔC_T_). The C_T_ value of each miRNA was normalized to the average value of endogenous genes (SnoRNA135 and U87) using the comparative cycle threshold method [48]. To compare differences between groups, differentially expressed miRNAs were identified by using SAM (two class, unpaired test, 100 permutations) on normalized C_T_ data [49] miRNAs with a FDR lower than 4%. Fold changes larger than 2 were considered as significant and were further analyzed for their potential targets. For mRNA data analysis, the C_T_ value of each mRNA was normalized to the average value of endogenous genes (*Hprt1, Rplp1* and *Actb*) using the comparative cycle threshold method [48]. For mRNA gene expression data analysis, differential expression was calculated using the 2^−ΔΔCt^ method as explained in the real-time PCR manual of Applied Biosystems. To compare the fold changes a student’s *t*-test was performed. An α level of 0.05 was selected for significance for all statistical tests.

## Results

### Expression Profile of miRNAs in the Normal Rat Adult Cochlear Sensory Epithelium

The expression pattern of miRNAs in the sensory epithelium, the major target of acoustic trauma, has not been previously established for the normal rat adult cochlea (2–3 months). Therefore, we screened the expression of 378 miRNAs using quantitative real time PCR (qRT-PCR) array. This set of miRNAs was examined because they represent comprehensive coverage of the Sanger miRBase and because many of them have been highly characterized in non-cochlear tissues.

We first examined the expression of 5 reference genes (U6, U87, SnoRNA135, SnoRNA202 and Y1). Among the reference genes, only U6 has previously been reported in cochlear tissue [36]. We found that 4 of the reference genes (U6, U87, SnoRNA135 and Y1) were highly expressed, whereas 1 (SnoRNA202) was undetectable. We also analyzed the stability of the expressed genes in the cochlear tissue after acoustic trauma using the NormFinder software algorithm (Andersen et al., 2004). Two highly expressed reference genes (U87and SnoRNA135) had low stability values of 0.007 and 0.010, indicating stable expression and therefore, the arithmetic mean of their threshold cycle (C_T_) values was used to normalize the expression levels of the miRNAs.

For the normal cochlear sensory epithelium, we performed a total of eight biological replications of miRNA profiling. These replications were divided into two groups for analysis as they were examined at two time periods. After applying the cut-off criterion of C_T_ ≤34, 208 miRNAs were detected in the first 4 samples and 212 miRNAs were detected in the remaining 4 samples out of the total 8 biological replicates. Among the detected miRNAs, a total of 176 miRNAs were expressed in all 8 samples and their expression levels were normalized to the average expression levels of the reference genes to generate the normalized C_T_ values (ΔC_T_) (Table 1). Out of these 176 genes, let-7b, 7e, miRs 200c, 24, 186, 154, 191 and 31 were highly expressed (ΔC_T_ values ≤3). The remaining miRNAs had diverse expression levels and their ΔC_T_ values ranged from 3.2 to 13.3. These results revealed the constitutive expression of miRNAs in the cochlear sensory epithelium. Importantly, many of the identified miRNAs have not been previously reported to be expressed in cochlear tissues.

**Table 1:** miRNA expression levels (ΔC_T_) for the normal group relative to endogenous gene expression.

miRNA	Expression Level (ΔC_T_) (mean±SD)	miRNA	Expression Level (ΔC_T_) (mean±SD)	miRNA	Expression Level (ΔC_T_) (mean±SD)	miRNA	Expression Level (ΔC_T_) (mean±SD)
**miR-200c**	0.6±0.9	miR-210	6.6±3.6	miR-126-5p	8.6±1.1	miR-10a	10.8±2.9
**let-7b**	2.0±0.9	miR-667	6.6±1.6	miR-193b	8.6±1.7	miR-324-5p	10.8±1.9
**miR-24**	2.3±1.2	miR-340-3p	6.7±0.9	miR-337-5p	8.7±0.9	miR-224	10.8±0.9
**let-7e**	2.6±0.7	miR-29b	6.7±1.4	rno-miR-1	8.7±1.4	miR-197	11.0±0.9
**miR-186**	2.6±0.8	miR-134	6.7±1.2	miR-135b	8.8±2.0	miR-331-5p	11.1±1.6
**miR-154**	2.7±0.9	miR-484	6.7±2.1	miR-151-3p	8.8±1.8	miR-339-5p	11.1±2.5
**miR-191**	2.9±1.1	miR-19b	6.7±1.8	rno-miR106b	8.9±1.5	miR-377	11.1±1.6
**miR-31**	3.0±0.7	miR-708	6.8±1.3	miR-106b	9.0±1.5	miR-376a	11.1±1.0
**miR-200a**	3.2±0.9	miR-155	6.8±0.8	miR-23b	9.0±1.1	rno-miR-224	11.2±1.1
**let-7d**	3.4±1.1	miR-434-3p	6.9±1.3	miR-214	9.0±1.1	miR-129-5p	11.2±1.2
**miR-204**	3.4±1.2	miR-184	6.9±0.7	miR-532-3p	9.0±0.9	miR-685	11.2±0.9
**miR-148a**	3.6±1.1	miR-124	6.9±0.8	miR-411	9.1±1.1	miR-185	11.2±1.4
**miR-145**	3.7±1.5	miR-423-5p	7.0±1.3	miR-205	9.1±0.9	miR-434-5p	11.3±1.3
**miR-29a**	4.0±1.4	miR-141	7.0±1.3	miR-130a	9.2±1.1	miR-450a	11.3±0.9
**miR-30e**	4.0±1.3	miR-96	7.1±1.6	miR-132	9.2±2.7	miR-190b	11.3±1.3
**miR-17**	4.1±1.6	miR-15b	7.4±1.5	miR-329	9.2±0.2	miR-345-3p	11.6±1.7
**miR-152**	4.1±1.1	miR-16	7.4±1.1	miR-18a	9.2±0.8	miR-129-3p	11.6±0.7
**miR-29c**	4.1±1.8	miR-328	7.5±1.5	miR-203	9.3±1.1	miR-598	11.6±0.7
**miR-125a-5p**	4.3±0.8	miR-125a-3p	7.5±1.1	miR-379	9.4±1.5	miR-505	11.7±0.9
**miR-99a**	4.9±1.4	miR-34b-3p	7.6±0.8	miR-331-3p	9.4±1.1	miR-369-3p	11.8±1.7
**miR-92a**	4.9±1.1	miR-195	7.6±3.9	miR-188-5p	9.4±1.6	miR-362-3p	11.9±1.5
**miR-182**	5.0±1.4	miR-1	7.6±1.6	miR-335-5p	9.5±1.4	miR-494	12.1±1.7
**miR-301b**	5.0±1.1	miR-100	7.7±1.4	miR-338-3p	9.6±1.3	miR-345-3p	12.1±1.3
**miR-34a**	5.1±4.5	miR-183	7.7±1.2	miR-135a	9.7±1.9	miR-451	12.1±1.7
**miR-30a**	5.1±0.8	miR-20b	7.8±1.3	miR-148b	9.7±1.7	miR-291a-3p	12.2±1.7
**miR-99b**	5.1±0.7	miR-146a	7.8±0.9	miR-221	9.7±1.7	miR-758	12.3±1.8
**let-7g**	5.2±1.2	miR-429	7.9±0.9	miR-296-5p	9.7±1.0	miR-496	12.4±1.6
**miR-106a**	5.3±5.2	miR-136	7.9±0.9	miR-192	9.7±0.4	miR-376b	12.4±2.1
**miR-181a**	5.4±1.2	miR-139-5p	8.0±0.4	miR-375	9.8±0.8	miR-450a-5p	12.5±1.6
**miR-200b**	5.4±0.9	miR-320	8.0±1.3	miR-384-3p	9.9±1.3	miR-682	12.5±1.1
**miR-150**	5.5±0.6	miR-25	8.0±0.9	miR-133b	9.9±2.7	miR-7a	12.5±1.0
**let-7i**	5.5±1.1	miR-103	8.1±2.1	miR-872	9.9±1.3	miR-668	12.6±1.7
**miR-30c**	5.6±1.2	miR-199a-5p	8.1±2.3	miR-333	10.0±0.7	miR-344-3p	12.8±1.7
**miR-199a-3p**	5.6±1.8	miR-433	8.2±0.9	miR-495	10.0±1.8	miR-350	12.8±1.6
**miR-125b-5p**	5.6±0.7	miR-19a	8.2±1.2	miR-128a	10.0±1.3	miR-544	13.2±2.3
**miR-26a**	5.7±1.2	miR-301a	8.2±1.2	miR-335-3p	10.1±0.8	miR-802	13.3±1.7
**miR-9**	5.7±1.2	miR-146b	8.2±0.9	miR-137	10.1±1.4	miR-544	13.2±2.3
**miR-30b**	5.8±1.2	miR-325	8.3±1.2	miR-190	10.2±1.3	miR-802	13.3±1.7
**miR-138**	5.9±1.7	miR-384-5p	8.3±1.2	let-7f	10.3±4.7	miR-802	13.3±1.7
**miR-126-3p**	5.9±0.7	let-7a	8.3±0.9	miR-376c	10.3±1.2	miR-544	13.2±2.3
**miR-130b**	6.0±1.9	miR-20a	8.3±1.7	miR-15a	10.4±2.2	miR-802	13.3±1.7
**miR-133a**	6.1±1.3	miR-142-3p	8.3±5.5	miR-187	10.4±1.5		
**miR-382**	6.3±4.1	miR-672	8.4±2.5	miR-107	10.7±1.1		
**miR-20b-3p**	6.5±1.2	miR-140	8.5±1.2	miR-323-3p	10.7±1.2		
**miR-27b**	6.5±1.5	miR-127	8.5±1.6	miR-339-3p	10.7±1.7		
**miR-26b**	6.6±1.5	miR-744	8.5±1.1	miR-324-3p	10.8±1.3		

**NOTE**: miRNAs are ranked based on their expression levels.

### Noise-induced Hearing Loss and Hair Cell Damage

**Figure 1:**
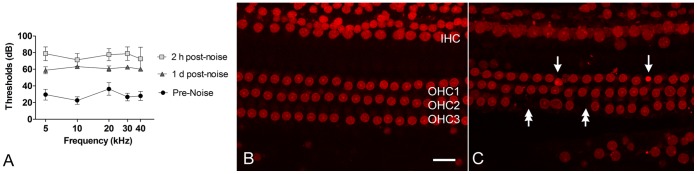
Loss of auditory function and the generation of sensory cell apoptosis following exposure to noise. (**A**) Comparison of the ABR thresholds (dB, mean ± SD) measured at three time points: pre-noise exposure and 2 h and 1 d post-noise exposure. The thresholds at the five tested frequencies, 5, 10, 20, 30 and 40 kHz, are presented. (**B**) A typical image of nuclear staining with propidium iodide in a section of the sensory epithelium from a control cochlea without exposure to noise. OHC1, OHC2 and OHC3 denote the first, second and third rows of OHCs. Bar: 20 µm. (**C**) Noise-induced morphological changes in sensory cell nuclei in the upper first cochlear turn of the sensory epithelium from a cochlea examined at 1 d post-noise exposure. Similar changes were noted at 2 h post-noise exposure (data not shown). Single arrows point to condensed OHC nuclei showing an increase in staining intensity with or without a decrease in nuclear size. Nuclear condensation is a sign of apoptosis. Double-arrows indicate the areas of missing cells.

To provide a context for the interpretation of the miRNA data after acoustic trauma, we examined the impact of noise exposure on cochlear function and morphology. Auditory brainstem response (ABR) thresholds were measured before (n = 8) and at 2 h (n = 4) and 1 d (n = 8) post-noise exposure to determine the functional status of the cochlea. Relative to pre-noise thresholds, we found threshold shifts of 47.12±4.3 dB and 32.3±6.2 dB (mean ± SD) at 2 h and 1 d post-noise exposure, respectively (Fig. 1A). A two-way ANOVA (time × frequency) revealed that the shifts were statistically significant for the time factor (F = 337.3; df = 2, 60; *p*<0.0001, Tukey: *p*<0.05). These results indicate that the noise exposure used in the current investigation induced significant cochlear dysfunction with similar magnitudes over the five tested frequencies.

We then examined the cochlear pathology to determine the magnitude of sensory cell damage during the acute phases at 2 h and 1 d post-noise exposure. Propidium iodide was used to reveal the nuclear morphology of the sensory cells. In the normal control cochleae (n = 4), we found no malformed nuclei in the sensory cells (Fig. 1B). In contrast, nuclear condensation indicated by an increase in the propidium iodide fluorescence and a decrease in nuclear size were observed in sensory cells of the upper first cochlear turn in the noise-traumatized cochleae at 2 h (data not shown) and 1 d (Fig. 1C) post-noise exposure. We also found areas of missing nuclei in the noise-traumatized cochleae at 1 d post-noise exposure (Fig. 1C), which is indicative of complete degradation of the nuclei. The numbers of damaged sensory cells (apoptotic and missing) accounted for 1.89% ±0.67% and 2.38% ±2.54% (mean ± SD) of the total number of sensory cells at 2 h and 1 d post-noise exposure, respectively. These changes were more prominent in the middle and basal turns of the cochlea. This level of sensory cell damage was comparable to our previous results obtained from the same rat model that was exposed to a similar level of noise [39]. Our previous investigation had shown that sensory cells exhibiting condensed nuclei had increased caspase-3 activity and positive Terminal deoxynucleotidyl transferase mediated dUTP Nick End Labeling, [19], [20] indicating that these cells were dying through the process of apoptosis. Together, our pathological data indicate that exposure to 120 dB noise induces sensory cell degeneration via the process of apoptosis and that apoptotic activity begins at 2 h post-noise exposure and continues for at least 1 d.

### Noise Exposure Induces a Time-dependent Expression Change in miRNAs in the Acute Phase of Cochlear Pathogenesis

**Figure 2:**
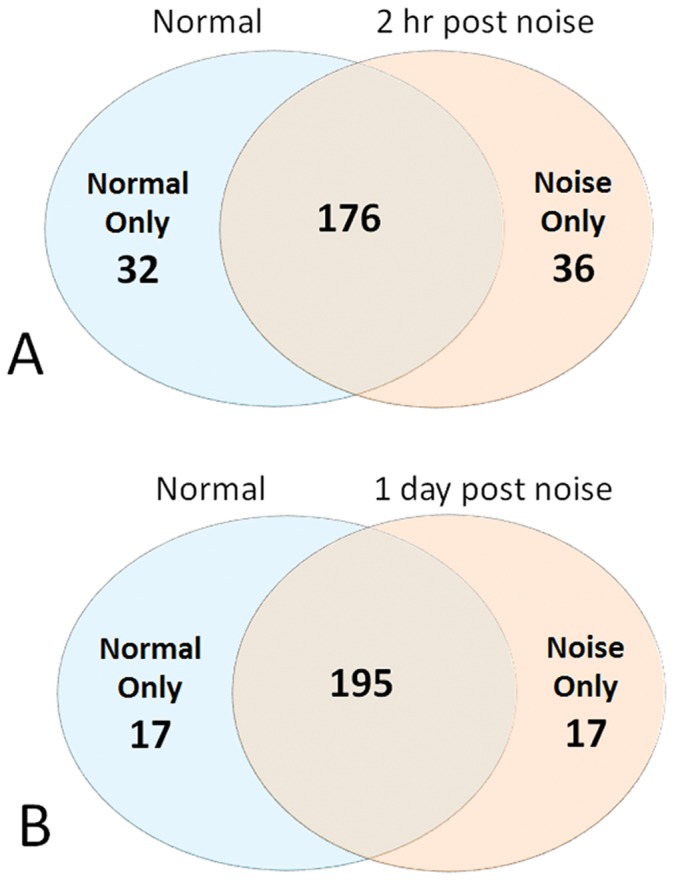
Venn diagram showing the number of miRNAs detected in either noise-exposed cochleae, control-cochleae, or both. (**A**) The numbers of miRNAs detected in the normal samples (pre-noise exposure), the noise-samples (collected at 2 h post-noise exposure) and both. Cut off C_T_ value ≤34. (**B**) The numbers of miRNAs detected in the normal samples (pre-noise exposure), the noise-samples (collected at 1 d post-noise exposure) and both. Cut off C_T_ value ≤34.

We examined changes in the expression of the miRNAs at two time points (2 h and 1 d, n = 4) after noise exposure. At 2 h post-noise exposure, 212 miRNAs were identified as expressed using the cut-off limit of C_T_ ≤34. The number of expressed miRNAs was slightly higher than that in normal control ears (212 vs. 208). The individual genes that were detected in the 2 h post-noise exposure group and the normal group were not identical (Fig. 2A). All of the miRNAs that were exclusively expressed in either the control or the noise-injured group had low or inconsistent expression patterns. This inconsistency may have biological significance in determining individual variations in cochlear responses to acoustic trauma. However, because of the limited sample size, we were unable to derive conclusive results from the statistical analysis. As a result, the current study focused on the miRNAs that were consistently expressed in both the noise and control samples. Using the algorithm of the significance of analysis of microarrays (SAM) to analyze expression changes after noise exposure, we found that 40 genes showed changes that were greater than a 2 fold increase or decrease (18 upregulated and 22 downregulated). However, only 1 gene, miR-331-5p was significantly upregulated at 2 h post-noise exposure (3.5 fold increase by SAM analysis, false discovery rate (FDR) = 0%). None of the miRNAs were significantly downregulated at this time point.

At 1 d post-noise exposure, 213 miRNAs were detected, similar to the number of detected genes reported for the 2 h noise group (212 miRNAs). Again, certain genes were exclusively expressed in either the control or the noise group (Fig. 2B); many of them were poorly expressed or had large individual variations. Due to the large variations in the data and the limited sample size, we were unable to derive conclusive results from the statistical analysis. We, therefore, focused our attention on the miRNAs that were consistently expressed in both the noise and control samples.

Using the SAM algorithm to analyze the expression changes, we found a downregulation-dominated change at 1 d post-noise exposure. None of the miRNAs were significantly upregulated at this time point. The expression level of miR-331-5p, which was upregulated at 2 h post-noise exposure, returned to a baseline level at 1 d post-noise exposure. Moreover, 20 miRNAs (miRs 10a, 107,124,130b, 146b, 183, 186, 190b, 194, 200c, 30d, 30e, 325, 333, 339-3p, 381, 429, 532-3p, 674 and 99b) were significantly downregulated with a fold change equal to or greater than 2.5 (Table 2, SAM analysis, FDR <4%). This downregulation dominated change in expression was not observed at 2 h post-noise exposure, suggesting that the noise-induced miRNA expression changes are time-specific and involve different sets of miRNAs at different time points.

**Table 2:** Fold Changes in miRNA Expression Following Acoustic Overstimulation.

2 h post-noise: Upregulation		1 d post-noise: Downregulation			
miRNA	Fold Change	miRNA	Fold Change	miRNA	Fold Change
**miR-331-5p**	3.50	miR-325	2.83	miR-532-3p	3.34
		miR-194	2.85	miR-130b	3.37
		miR-30d	2.85	miR-429	3.38
		miR-333	2.87	miR-190b	3.38
		miR-339-3p	2.87	miR-124	3.38
		miR-99b	2.87	miR-186	3.39
		miR-674	2.88	miR-30e	3.46
		miR-183	2.88	miR-381	4.03
		miR-146b	2.89	miR-107	4.84
		miR-200c	2.89	miR-10a	4.87

NOTE: Significance of analysis of microarrays (SAM), False discovery rate (FDR) <0% at 2 h, FDR<4% at 1 d.

### Bioinformatic Analysis Reveals Potential Targets for miRNAs

miRNAs regulate cellular functions by modulating their targeted mRNAs. We, therefore, analyzed potential targets of the expression-altered miRNAs using bioinformatic software and databases. First, the potential target genes for each miRNA were identified using TargetScan. Among the 21 miRNAs that exhibited expression changes after noise exposure (1 miRNA upregulated at 2 h and 20 miRNAs downregulated at 1 d), 5 miRNAs (miRs 532, 331-5p, 333, 339-3p and 674) were not included in the TargetScan database and were excluded from the analysis. Nine miRNAs (miRs 30d, 30e, 99b, 107, 130b, 146b, 190b, 200c and 429) were found to have limited numbers of target genes to be sufficient for subsequent functional annotation and therefore, were excluded from further analysis. For the remaining 7 miRNAs (miRs 10a, 124, 183, 186, 194, 325 and 381), each was found to have a list of target genes, and these identified genes were classified into functional clusters using the database for annotation, visualization and integrated discovery (DAVID). The functional clusters were then processed for the generation of enriched gene ontology (GO) terms.

**Figure 3:**
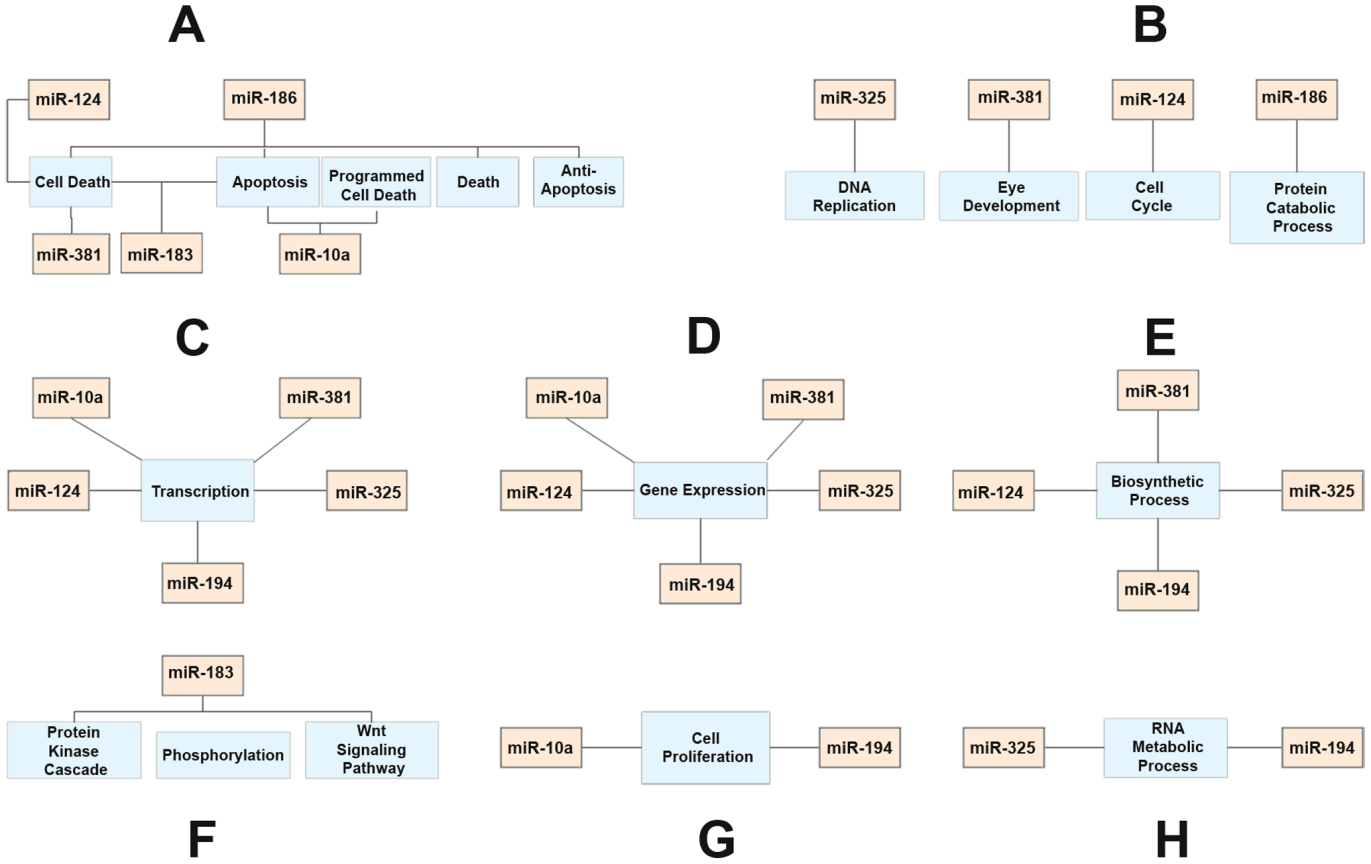
Schematic of miRNAs and their associated gene ontology (GO) biological process terms. (**A**) miRNAs associated with cell death related GO terms. (**B**) miRNAs associated with DNA replication, eye development, cell cycle and protein catabolic process GO terms. (**C**) miRNAs associated with transcription GO terms. (**D**) miRNAs associated with gene expression GO terms. (**E**) miRNAs associated with biosynthetic process GO terms. (**F**) miRNAs associated with protein kinase cascade, phosphorylation and Wnt signaling pathway GO terms. (**G**) miRNAs associated with cell proliferation GO terms and **(H**) miRNAs associated with RNA metabolic process GO terms.

Each miRNA was found to be associated with a list of biological processes. Based on the *p* values (modified Fisher exact test), we selected the five processes that had the highest association with their corresponding miRNA. As noted in Figure 3, cell death and apoptosis are the major cellular processes shared by the majority of the miRNAs. Other biological processes include transcription, cell proliferation, gene expression, biosynthetic process, cell cycle, phosphorylation, protein catabolic processes, RNA metabolic processes, DNA replication and eye development, as well as signaling via the Wnt pathway and protein kinase cascades. These analyses suggest that cell death and apoptosis are two of the major cellular processes that are associated with the changes in expression of miRNAs.

**Figure 4:**
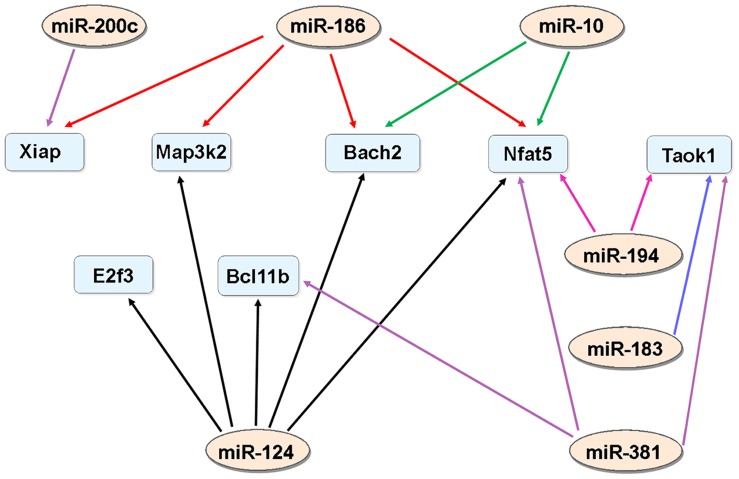
miRNA/mRNA targeting pathway map. Targeting interactions between a total of seven miRNAs (light pink ellipses) and seven mRNAs (light blue rectangles) are depicted. These seven miRNAs which were downregulated 1 d post-noise exposure were found to have a list of target genes in the TargetScan database. The remaining 14 genes (13 downregulated at 1 d post-noise exposure and 1 upregulated at 2 h post-noise exposure) were either not included in the TargetScan database or had limited number of target genes to be sufficient for subsequent target analysis.

The apoptosis findings prompted us to focus our subsequent analysis on the cellular processes related to cell death. To narrow down the scope of our target gene search, we selected only the genes (related to cell death) that were targeted by at least 2 miRNAs. With this criterion, we identified 7 genes (*Nfat5, Bcl11b, Bach2, Xiap, E2f3, Taok1* and *Map3k2*) for the 7 miRNAs (miRs 10a, 124, 183, 186, 194, 200c and 381; Fig. 4). The bioinformatic analysis revealed the potential target genes for miRNAs and these target genes might be involved in the regulation of apoptotic activity in noise-damaged cochleae. Moreover, the 7 miRNAs, which do not belong to the same family of miRNAs, shared common targets, suggesting that these miRNAs may work together to regulate target expression. Conversely, this analysis confirms that multiple miRNAs can target a single predicted gene.

### Experimental Validation Reveals Taok1 as a Potential Target of miRNAs

**Figure 5:**
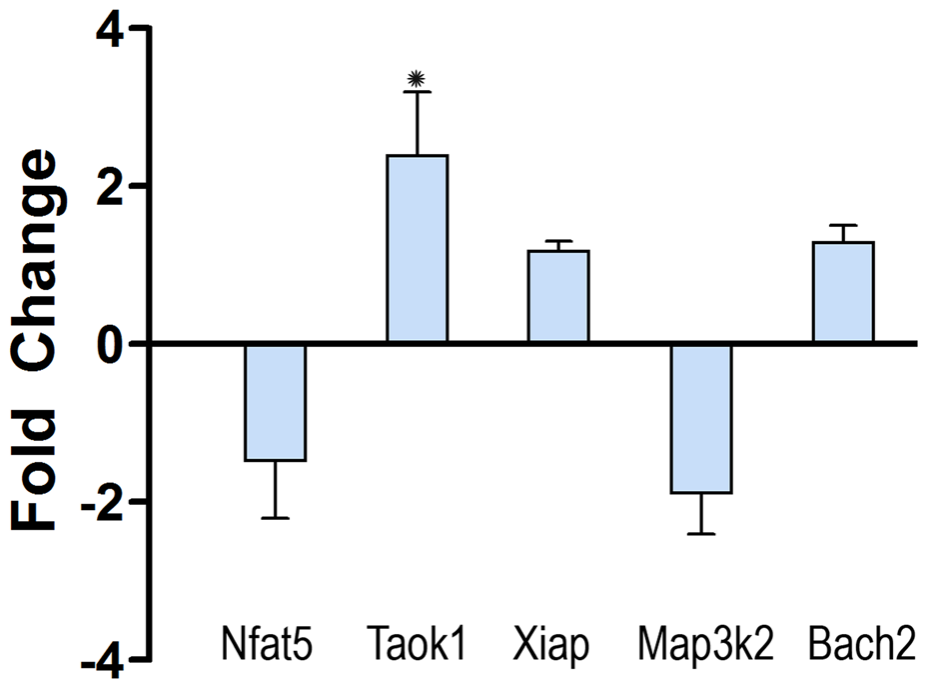
Changes in expression levels of five target mRNAs examined 1 d after noise exposure. *Nfat5* and *Map3k2* are downregulated and *Taok1*, *Xiap* and *Bach2* are upregulated in the noise-exposed cochleae compared to the normal cochleae. *p<0.05, Student’s *t*-test.

The bioinformatic analysis of miRNA targets for the 1 d group data identified seven genes (*Nfat5, Bcl11b, Bach2, Xiap, E2f3, Taok1* and *Map3k2*) as potential regulators of noise-induced cell death. To provide experimental verification of this analysis, 5 predicted mRNA targets (*Nfat5*, *Taok1*, *Xiap*, *Map3k2* and *Bach2*) were experimentally examined for transcriptional changes following acoustic trauma using qRT-PCR. In the normal cochleae (n = 4), all the examined genes were highly expressed at levels close to the reference genes (ΔC_T_ ≤3). At 1 d post-noise exposure (n = 4), *Taok1*, a predicted target of miR-183, was significantly upregulated (Student’s *t*-test, *p*<0.05) by 2.3-fold compared to normal (Fig. 5). In contrast, the remaining four genes exhibited no significant change in their expression levels (<2 fold change).

**Figure 6:**
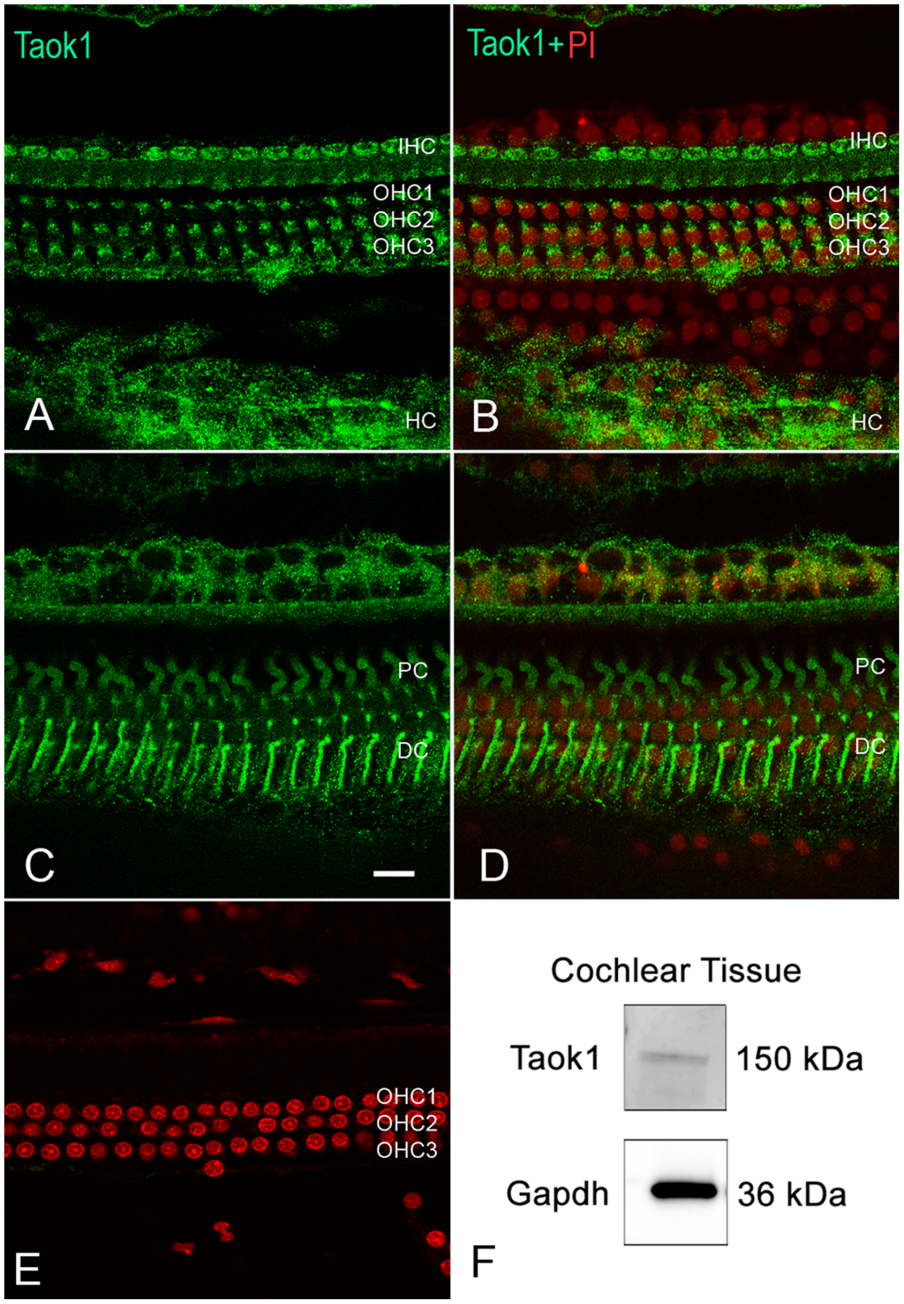
Immunolabeling of Taok1 protein in the cochlear sensory epithelium. (**A**) A typical image taken using confocal microscopy showing Taok1 immunoreactivity (green fluorescence) in IHCs, OHCs, and Hensen cells (HC). (**B**) The merged image from (A) and nuclear staining (propidium iodide, red fluorescence) in the same region. (**C**) Image showing Taok1 immunoreactivity in pillar cells (PC) and Deiters cells (DC). Bar: 10 µm. (**D**) The merged image from (C) and nuclear staining in the same region. (**E**) The image of negative control tissue tested without the primary antibody exhibiting nuclear propidium iodide staining in the OHCs with no clear green fluorescence in the same region. (**F**) Western blot analysis of Taok1 expression in the rat cochlea. Gapdh was used as a cochlea tissue-specific loading control. A single band around 150 kDa corresponds to Taok1 and the 36 kDa band corresponds to Gapdh.

To determine whether the Taok1 protein was expressed in the cochlear sensory epithelium, we performed immunolabeling for Taok1 (n = 3). In the organ of Corti, Taok1 immunoreactivity was found in the cytoplasm of both inner hair cells (IHC) and outer hair cells (OHC) (Fig. 6A and 6B). Immunoreactivity was also observed in the supporting cells of the cochlear sensory epithelium, including the pillar cells and Deiters cells (Fig. 6C and 6D) that have a direct contact with the hair cells. Negative control tissues tested without the primary antibody exhibited no clear fluorescence (Fig. 6E). To verify the specificity of the immunolabelinΔg, we verified the molecular weight of the protein targeted by the Taok1 antibody using a western blotting assay (n = 3) and found a band at 150 kDa (Fig. 6F), consistent with the reported molecular weight of the protein in non-cochlear tissues [50], [51]. Gapdh (36 kDa) served as a tissue specific loading control (Fig. 6E). Together, these findings suggest that *Taok1* is a potential target of miRNAs in the cochlear sensory epithelium.

### Inhibition of miR-183 in Cochlear Organotypic Culture Leads to Upregulation of Taok1

To further confirm the interaction between *Taok1* and miRNAs, we tested if inhibition of miR-183, a potential Taok1-modulator that was downregulated after the noise exposure, altered the expression level of *Taok1*. Because miRNA-183 has other potential mRNA targets, we examined two of these, *Egr1* and *Irs1,* to verify whether inhibition of miR-183 was able to alter expression of targets other than *Taok1*. To this end, we transfected organ of Corti explants from P3 rat cochleae with a morpholino oligo that was complementary to miR-183.

**Figure 7:**
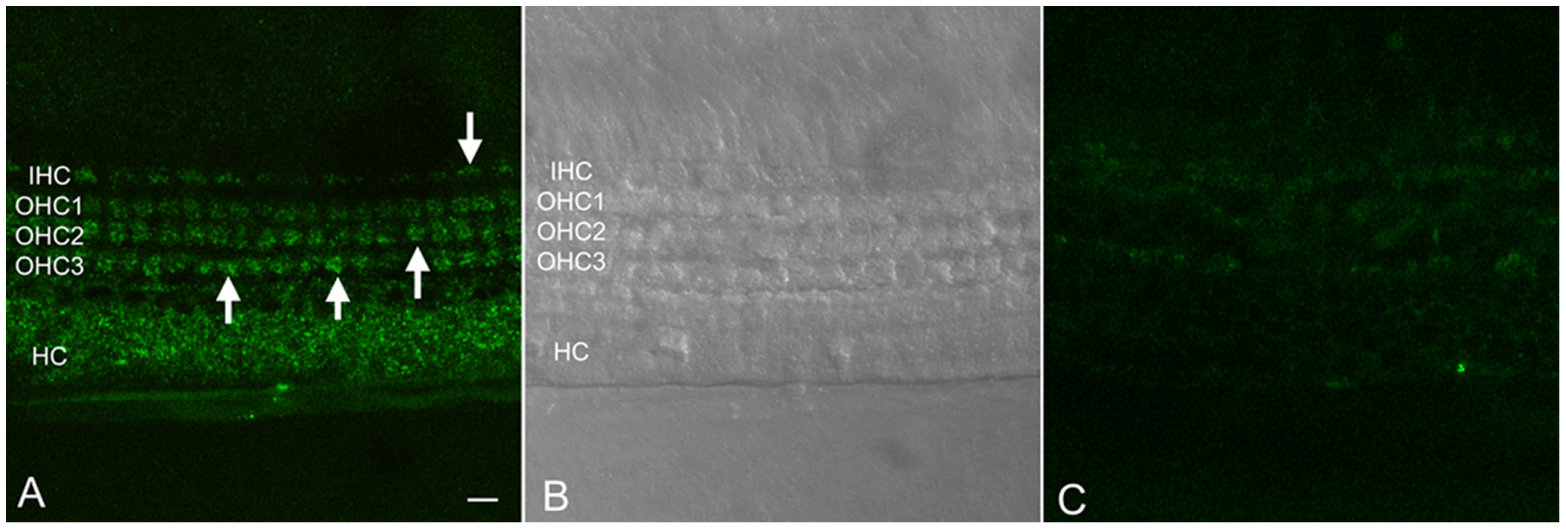
Typical images of cochlear organotypic explants treated with fluorescein-conjugated morpholino oligo. (**A**) A typical cross-section image of an organotypic cochlear explant cultured with fluorescein-conjugated morpholino (24 h) taken using confocal microscopy. Arrows indicate the presence of the fluorescein-conjugated morpholino oligo (green fluorescence) in the cytoplasm of the cells. OHC1, OHC2 and OHC3 indicate the first, second, and third rows of OHCs. HC indicates Hensen cells. (**B**) DIC image showing the three rows of OHCs, the single row of IHC and the HC in the same region as (A). (**C**) A typical cross-section image of an organotypic cochlear explant cultured without fluorescein-conjugated morpholino (24 h) to demonstrate the level of auto-fluorescence. Scale bar: 10 µm.

We first tested if the morpholino oligo could be transfected into cochlear explants (n = 4) using a fluorescein-tagged morpholino oligo (5 µM). At 24 h post-transfection, fluorescein labeling was observed in the cytoplasm of the OHC and IHC, as well as in the Hensen cells (Fig. 7A), indicating entry of the oligo into the target cells (Fig. 7B). The control explants with no transfection did not exhibit fluorescein labeling (Fig. 7C). According to the manufacturer, a 5 µM concentration of the oligo is sufficient to inhibit miRNA function. Thus, all subsequent transfection experiments were performed at a concentration of 5 µM.

**Figure 8:**
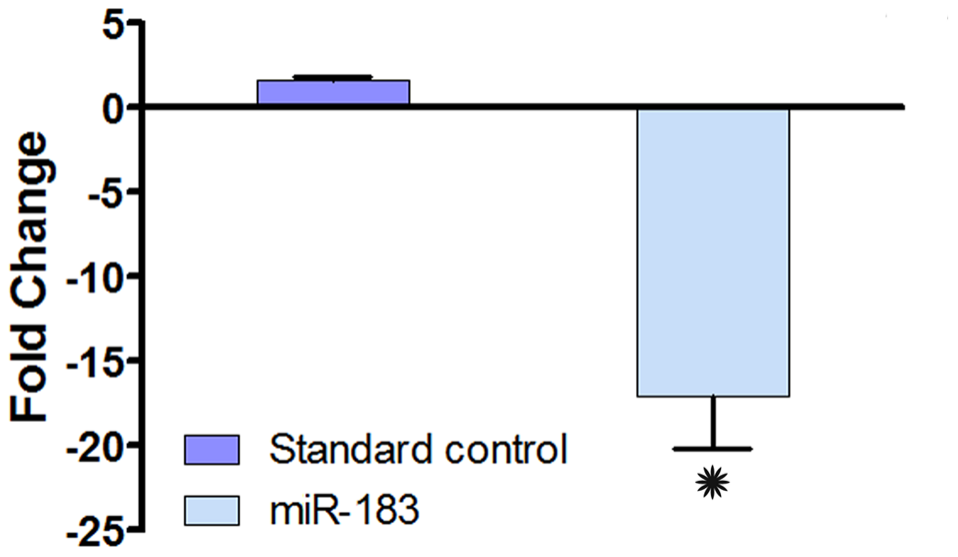
Downregulation of miR-183 in cochlear organotypic cultures treated with antisense morpholinos. Changes in miR-183 expression in the cochlear explants treated with miR-183^*^ antisense morpholino. *p<0.05, Students *t-*test.

Cochlear explants (n = 3) were subsequently transfected with the miR-183* morpholino, to suppress the endogenous miRNA; miR-183. At 24 h post transfection, the expression level of miR-183, determined by qRT-PCR, was significantly reduced compared to the control explants transfected with scrambled control oligos (Fig. 8; Student’s *t*-test, *p*<0.05). This result confirms that the morpholino inhibited endogenous miRNA expression. Morphological inspection of both transfected and non-transfected explants revealed that the organ of Corti maintained its normal structural integrity, indicating that both the scrambled and active miRNA morpholinos did not affect cell survival in the transfected explants (data not shown).

**Figure 9:**
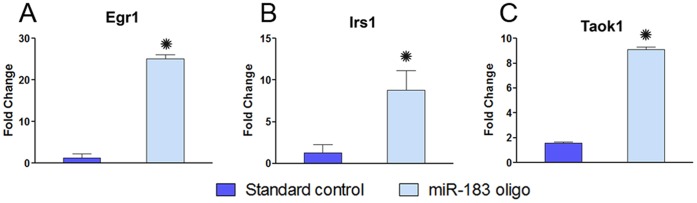
Expression levels of miRNA target genes in cochlear organotypic cultures treated with antisense morpholinos. Expression changes in (A) *Egr1*, (B) *Irs1* and (C) *Taok1* after miR-183* transfection. *p<0.05, Students *t*-test.

To further verify whether inhibition of miR-183 altered the expression of its associated target; we next examined the change in the *Taok1* target expression. Now, *Taok1* was the only target gene identified from our previous bioinformatic analysis (which included a narrow selection of only those genes which were targeted by at least 2 miRNAs). To verify whether inhibition of miR-183 altered expression of targets other than *Taok1*, we broadened the scope of our analysis to include additional targets of miR-183 consisting of *Egr1* and *Irs1*. Our results indicated that compared to the control cultures transfected with the scrambled oligo, the explants transfected with the miR-183 oligo exhibited significant upregulation of all 3 targets, *Egr1, Irs1*and *Taok1* (Fig. 9A, 9B and 9C; Student’s *t-*test, *p*<0.05). These observations support the inverse relationship between miR-183 and its associated targets, including *Taok1*.

## Discussion

Identification of constitutively expressed miRNAs in specific tissues has been a major focus of miRNA investigations [52]–[56]. While numerous studies have profiled miRNA expression in mouse inner ears, miRNA expression in the rat cochlea has not been examined. Here, we successfully detected a group of miRNAs in both normal and noise-exposed sensory epithelia in the rat cochlea.

The current study reveals constitutive expression of miRNAs in the normal cochlear sensory epithelium and noise-induced changes in the expression of those miRNAs during the acute phase of cochlear pathogenesis. The changes in expression are time-dependent. Bioinformatic analysis identifies the potential target genes with apoptotic properties for the seven significantly-downregulated miRNAs that have been well characterized. Further expression analysis of the predicted target genes reveals an inverse relationship between the expression levels of miR-183 and *Taok1*. This relationship was further confirmed by manipulation of miR-183 expression in cochlear organotypic cultures. Together, these observations implicate miRNAs as potential players in the regulation of cochlear responses to acoustic trauma.

Previously, Weston et al. (2006) screened the expression of 344 mature miRNAs in the whole inner ear of the developing mouse (P0–P100). Of the 344 mature miRNAs, 102 miRNAs were expressed from P0–P36. Many of the identified miRNAs were subsequently confirmed in a recent investigation by Wang et al. (2010), who showed the expression of 122 miRNAs at E13.5 and 199 miRNAs at E16.5. More recently, Elkan-Miller et al. (2011) examined the expression of 586 miRNAs and detected 138 miRNAs in the P2 mouse cochlea. Here, we examined the expression of 378 miRNAs and detected 176 miRNAs in the adult rat cochlear sensory epithelium. Among the 178 miRNAs, miRs 135a, 136, 139-5p, 146a, 146b, 155, 184, 186, 187, 188-5p, 190b, 193b, 203, 210, 224, 291a-3p, 296-5p, 301a, 301b, 323-3p, 324-3p, 329, 34b-3p, 350, 369-3p, 375, 376a, 376c, 384-5p, 411, 433, 434-5p, 434-3p, 532-3p, 598, 667, 668, 672, 682, 708, 744, 758, and 872 have not been previously identified in a mouse model [36], [57], [58]. These miRNAs may be species specific for the rat cochlea. However, their detection may also be due to our enriched sensory epithelium samples compared to the whole cochlear samples used in previous studies, or due to the higher sensitivity of the qRT-PCR technique used in our miRNA assessment compared with the microarray analysis used in previous investigations [59], [60].

We identified miRNAs 182, 183 and 96 in the rat, which are three intensively investigated miRNAs that are present in mouse ears [61], [62]. Thus, this cluster of miRNAs seems to be conserved between species. We found a significant downregulation of miR-183 at 1 d post-noise exposure. However, the expression levels of the other 2 miRNAs were not significantly altered. This differential expression pattern has been observed in previous studies and has been attributed, in part, to variation in the rate of miRNA degradation [63], [64]. Thus, there is a possibility that the lack of change in miRs-182 and -96 following acoustic trauma is due to a slower degradation rate or no degradation compared to the targeted degradation of miR-183, which in turn may lead to the inconsistent expression pattern of these miRNAs within the cluster.

In the present investigation, we found a time-dependent alteration in miRNA expression post-noise exposure. At 2 h post-noise exposure, only one miRNA exhibited a significant change in expression. In contrast, there was an increase in the number of miRNAs that were altered at 1 d post-noise exposure. This temporal pattern of changes in expression is likely to be related to the progression of sensory cell degeneration post-noise exposure. As we reported earlier, sensory cell lesions grow in a time-dependent manner [9], [11]. The growth of the lesion is expected to provoke more cells to undergo the degenerative process and consequently, more miRNAs to undergo changes in expression.

Another possible contributor to the temporal change in miRNA expression is differences in damaging initiators during the different phases of cochlear pathogenesis. Acute damage to cochlear tissues observed 2 h post-noise exposure is primarily associated with the mechanical stress caused by physical disturbances to the cochlear structure during the period of noise exposure. In contrast, subsequent pathologies, including energy exhaustion, [65] oxidative stress, [66] and ionic imbalance, [67]–[69] may be the result of a metabolic disruption. These metabolic disruptions are likely to cause changes in miRNA expression through different mechanisms than those caused by acute mechanical stress.

In our study, the majority of miRNAs detected at 1 d post-noise exposure were significantly downregulated when compared to their constitutive expression levels. This finding of a downregulation dominated change is consistent with previous observations of oxidative stress-related changes in miRNA expression in cultured cells derived from the organ of Corti [70] as well as in non-cochlear apoptotic models [71]–[73]. As miRNAs act as inhibitors of mRNA in controlling cellular processes [74]–[76] a reduction in miRNA expression following acoustic trauma may lead to an increase in the expression of mRNA targets.

Many of the target genes of miRNAs that underwent changes in expression, as revealed by our bioinformatic analysis, have been linked to sensorineural hearing loss. For example, *Xiap* is a predicted target of miR-186. Previous studies have shown the involvement of this target gene in protection against noise-induced hearing loss when it is over-expressed in transgenic mice [17], [77]. *Mapk*, a predicted target of miR-124, has been suggested to be involved in stress-related pathways in the auditory system [18], [78] and also to be linked to cochlear apoptosis induced by acoustic trauma [79]–[81]. *E2f3*, a predicted target of miR-124, was recently identified to be upregulated 2 h post-noise exposure in the chinchilla cochlea and was linked to the p38/MAPK signaling pathway [82]. *Bcl11b*, a predicted anti-apoptotic target of miRs 124 and 381, is required for cell survival, and inhibition of *Bcl11b* both *in vitro* and *in vivo* leads to apoptosis [83], [84]. Recently, *Bcl11b* was associated with age-related hearing loss and was suggested to be required for OHC survival and normal hearing [85]. Thus, miRNA/mRNA target pairs may be present in the cochlea and may be involved in regulating apoptosis-related pathways. To further our understanding of noise-induced apoptosis in the cochlea, it will be important to identify these miRNA/mRNA target pairs. Furthermore, it will be important to understand the biological significance of these target pairs and their relationship with sensory cell damage following noise exposure.

One of the possible miRNA/mRNA target pairs revealed by the current study is *Taok1/*miR-183. *Taok1* was identified by our bioinformatic analysis and its association with miR-183 was experimentally verified. *Taok1* contains two binding sites for miR-183 in its 3′untranslated region. Both sites are perfectly complementary to the miR-183 sequence in the miRNA seed region. In non-cochlear tissues, *Taok1* has been associated with activation of the mitogen-activated protein kinase pathway in response to stress and DNA damage [86]–[88]. In cancerous tissues, *Taok1* has been found to activate the c-Jun N-terminal kinase mitogen-activated protein kinase pathway [89]. In human neuroblastoma cells, Taok1 transfection induces apoptosis [90]. In noise-damaged cochleae, the mitogen-activated protein kinase pathway has been linked to cochlear apoptosis and inhibition of this pathway reduces apoptosis [91], [92]. These observations suggest the involvement of *Taok1* in the regulation of cochlear responses to acoustic trauma, possibly through the regulation of apoptosis. Bioinformatic analysis also revealed other targets of miR-183, including *Egr1* and *Irs1*. Previously, acoustic overstimulation (125 dB SPL) in the rat cochlea was found to increase the transcriptional expression of *Egr1*, which further led to an increase in its protein expression [93]. Irs1 modulates insulin signaling pathways and has not been previously identified in the cochlea. However, a previous study has shown Irs2-deficient mice to exhibit sensorineural hearing loss [94]. Thus, it would be important to study whether Irs1 also plays a role in regulating cochlear responses to acoustic trauma.

The current study documents the constitutive expression pattern of 176 miRNAs in the normal rat cochlear sensory epithelium and noise-induced changes in the expression of these miRNAs. The changes in expression are time-specific. Further target prediction analysis and subsequent experimental verification revealed the miR-183/*Taok1* target pair. These results implicate miRNAs as regulators of noise-induced cochlear responses to acoustic trauma. The discovery of differentially expressed miRNAs after noise exposure in the rat cochlea can assist with the future exploration of miRNA/mRNA target pairs that may be manipulated to reduce noise-induced cochlear damage.
